# Calculated left ventricular outflow tract diameter for critically ill patients

**DOI:** 10.1186/s40560-022-00623-6

**Published:** 2022-06-21

**Authors:** Eline G. M. Cox, Jacqueline Koeze, Iwan C. C. van der Horst, Renske Wiersema

**Affiliations:** 1 Department of Critical Care, University of Groningen, University Medical Center Groningen, Groningen, The Netherlands; 2 Department of Intensive Care Medicine, Maastricht University, Maastricht University Medical Centre+ Maastricht, Maastricht, The Netherlands; 3 Cardiovascular Research Institute Maastricht (CARIM), Maastricht University, Maastricht, The Netherlands

**Keywords:** Prospective study, Critical care ultrasound, Cardiac output, Left ventricular outflow tract, Critical care, Intensive care unit

## Abstract

A fast and reliable left ventricular outflow diameter (LVOTd) estimation may aid in quickly estimating cardiac output. However, obtaining a correct LVOTd can be difficult in intensive care patients, potentially leading to errors and a cardiac output deviation. In this study, the measured LVOTd was compared with the expected LVOTd when estimated using an existing formula in 1177 critically ill patients. We show that estimated LVOTd based on baseline data can aid when obtaining LVOTd is difficult or impossible and simplified estimation based on a formula may allow for more reliable and accessible measurement of cardiac output.

## Dear Editor,

Critical care ultrasonography (CCUS) in the intensive care unit (ICU) is increasingly applied [1]. One of the most frequently used measurements is cardiac output (CO), calculated from the velocity–time integral and left ventricular outflow tract diameter (LVOTd). Research has revealed that obtaining a correct LVOTd can be difficult in critically ill patients, potentially leading to errors and a CO deviation [2]. A fast and reliable LVOTd estimation may aid in quickly estimating CO. Therefore, we studied (a) the association between patient characteristics and LVOTd in critically ill patients and (b) an existing formula to estimate LVOTd [3].

We analysed data from two prospective observational cohorts (simple intensive care studies I and II (SICS-I [4] and SICS-II [5])) in which all acutely admitted adults with an expected ICU stay over 24 h were included. Trained researchers performed CCUS to obtain the parasternal long-axis view, and an independent expert performed the measurements. We used correlations (plots), the Pearson (*r*) coefficient, regression analysis and Bland–Altman plots to assess the data.

**Fig. 1:**
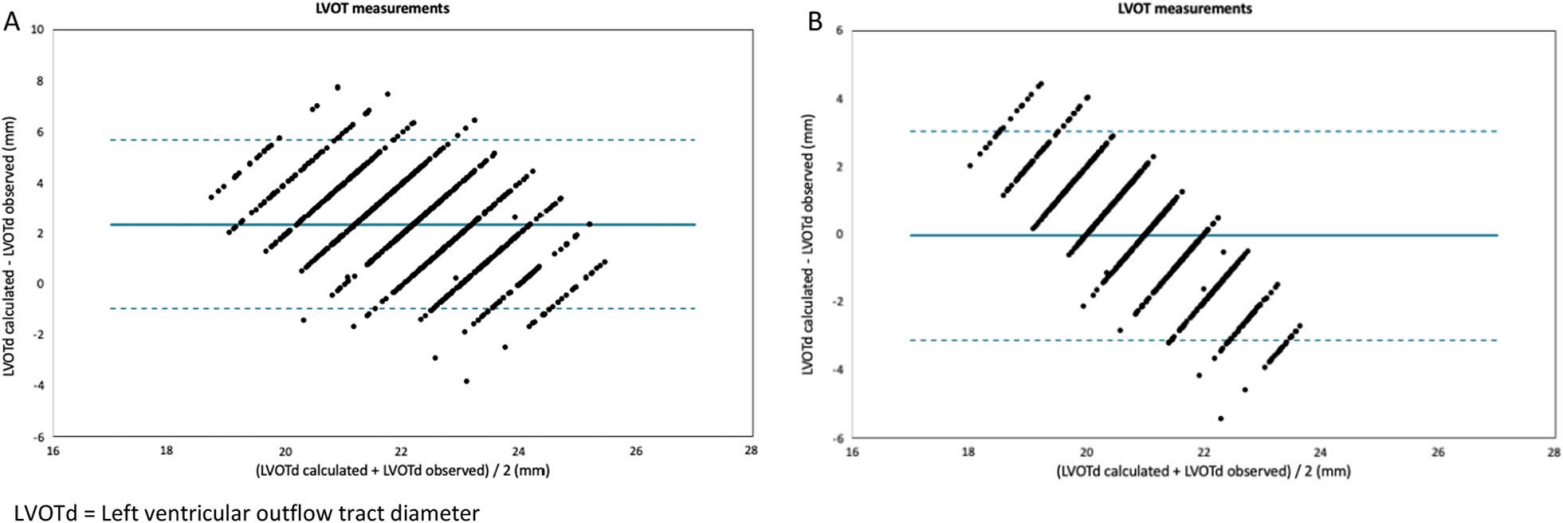
**A** Bland–Altman plot known formula and observed LVOTd. **B** Bland–Altman plot new proposed formula and observed LVOTd. *LVOTd* left ventricular outflow tract diameter

Patient inclusion took place from March 27th, 2015, to July 22nd, 2017 (SICS-I cohort) and March 14th, 2018, to July 10th, 2019 (SICS-II cohort). In total, 2208 unique patients were included, of which 1177 had validated LVOTd measurements and were included in the current analysis. Mean LVOTd was 21 mm (± 2 mm), with a significant difference between males (22 mm ± 2) and females (20 mm ± 2 mm, *p* < 0.001). LVOTd was associated with sex, height, weight, body surface area and body mass index. In the final multivariate regression, height, weight, and sex were significantly associated with LVOTd. The formula by Leye et al. correlated well but seemed to overestimate LVOTd systematically (mean difference 2.4 mm, ± 1.7 mm, LoA − 1.0 to 5.8 mm). Adjusting the intercept for the included population solved this issue (mean difference 0.0 mm, ± 1.7 mm, LoA − 3.4 mm to 3.4 mm). Figure 1 shows the observed LVOTd in our population and the expected LVOTd (a) when estimated using the formula by Leye et al. and (b) when the formula with adjusted intercept was used. Robustness of our findings was tested by internal bootstrap validation.

This analysis shows that using estimated LVOTd based on baseline data can aid when obtaining a parasternal long-axis view is difficult or impossible. LVOTd was, unsurprisingly, associated with height, weight and gender. The formula by Leye et al. correlated well but systematically overestimated LVOTd in our cohort, but adjusting the intercept corrected this issue for the studied population. This is the first study of its kind in acutely admitted critically ill patients with the present sample size, a population in which it may be specifically useful to estimate CO. Even though standardized LVOT will not directly impact therapeutic interventions, small errors in LVOTd measurement lead to major differences in CO because of the mathematical contribution of LVOTd in the formula. However, direct use of VTI is not influenced by LVOT and could also be used to reflect changes in stroke volume. To conclude, simplified estimation based on a formula may allow for more reliable and accessible measurement of CO in the critically ill. Population-based adjustment by calibration of the used formula may possibly further enhance accuracy.

## Data Availability

The datasets used and/or analysed during the current study are available from the corresponding author on reasonable request.

## Abbreviations

CCUS: Critical care ultrasonography
ICU: Intensive care unit
CO: Cardiac output
LVOTd: Left ventricular outflow tract diameter
SICS: Simple intensive care studies
LoA: Limits of agreement
